# Apoplexy

**Published:** 1890-03

**Authors:** 


### APOPLEXY.

In apoplexy, the person falls down as if suddenly struck with death. There is neither thought, feeling, nor voluntary motion. There is no sign of life, except that of deep heavy breathing. It comes on with the suddenness of the lightning's flash, and with as little premonition. If the person is not really dead, the face is flushed, the breathing loud, and the pulse full and strong, usually. In mild attacks, a person is found in bed of a morning apparently in a sound sleep; but no amount of shaking makes any impression. The earliest Greek writers described apoplexy with minute accuracy, which has scarcely been exceeded since, showing that it is a malady belonging to all time. To pass from apparent perfect health to instant death on entering one's own dwelling, or sitting down to the family table, or while at the happy fireside, in the loving interchange of affectionate offices, strikes us as being terrible. But the terror belongs to the witness; the victim is destitute of thought, feeling, sensation and consciousness. In many cases, after lying for hours, and even days, in a state of insensibility, the patient wakes up as if from an uneasy sleep or dream; but often, as many sadly know, there is no return to life again. The essential nature of the disease seems to be such an excess of blood in the brain that its appropriate vessels or channels cannot contain it, and it is "extravasated," let out, upon the substance of the brain itself, and thus arrests the functions of life.

Corpulent persons with short neck, are almost the sole subjects of apoplexy, but it may be induced by falls, blows, shocks, and over-doses of certain drugs. Apoplexy is an avoidable disease, except in some cases of accidents, which we can neither foresee nor prevent; it results from too much blood in the brain, which is either sent there too rapidly, or detained there in some unnatural manner, the effect being the same. Whatever "excites the brain," such as intense and long thought on one subject, all kinds of liquors; any drink containing alcohol, whether ale, beer, cider, wine, or brandy, induces apoplexy. So will a hearty meal, especially if alcoholic drinks are taken at the same time; going to bed soon after eating heartily, sleeping on the back, if corpulent, may bring on an attack any night; so will a hot bath, so will a cold bath soon after eating. The ultimate effects of all opiates are to *detain* the blood in the brain, while the things just mentioned send it there in excess. The great preventives are warm feet, regular and temperate habits, and the avoidance of opiates, tobacco, and all intoxicants. In case of an attack, send for a physician. Meanwhile, put the feet in *hot* water, and envelop the head with ice. It is safer to live in a hilly than level country, in town than country. Winter is more dangerous than summer. The liability increases rapidly after forty years of age, greatest at sixty, when it gradually diminishes. Statistics seem to show that the most dangerous years are from fifty to sixty.

